# Supplementary material for: Patterns of Intron Gain and Loss in Fungi
Source: PLoS Biol. 2004 Nov 30;2(12):e422. doi: 10.1371/journal.pbio.0020422 (PMC532390; doi:10.1371/journal.pbio.0020422)
Supplement: Table S1 — Also available at http://genes.mit.edu/NielsenEtAl/. (4.3 MB ZIP). [file pbio.0020422.st001.zip › NielsenEtAl/html/131.html]

AN4969.1.NCU03899.1.MG01027.1.FG09262.1


```
 CLUSTAL W (1.82) Multiple Sequence Alignments - Introns Inserted


Sequence 1: NCU03899.1	743 aa
Sequence 2: FG09262.1	726 aa
Sequence 3: MG01027.1	747 aa
Sequence 4: AN4969.1	738 aa
Alignment Length: 759 aa
Number Identitical Residues: 337 aa
Alignment Score (without introns) 16910


MG01027.1 	MSQDQ--GLWSVRRARE0TLGNLNLNSAIPQPPSAMKRSSSITGQGAPYTAGHSRSLSGS
NCU03899.1	MSQDSNAGLWSVRRPRE0TLHGVNLTSGIPAPPSTLKRQS-TIGT--IGTG-HGRSLSG-
FG09262.1 	MSQDT--GLWSVRRPRE0TLGGISANSAIPQPGSTMKRSSSNIGG--NYPGSHVRSMSGS
AN4969.1  	MSQDT--GLFSIKRPRE0TLGSVQNFSSLPQPSSALKRTSSIGYQNPPFTSQHTRSMSL-
          	****   **:*::*.** ** .:.  *.:* * *::** *:    ..  .. * **:*  

MG01027.1 	RQSLAMSRPSQPVFQRTSTGASAGEFGLSSVKR--PSSYQNHTSTFS--NSKGLTPGGRA
NCU03899.1	RQSLALPRPSQPLFQRSS------DIGLGSVKR--ASVHNMNTSQFKPYGTPGSSGIPRS
FG09262.1 	RHSLAMPRPSQPMFQRSSSGTNLVDLGLSSVKR--SSFAPKST------FTPG-TATKRA
AN4969.1  	--LNSVGRPQQPNFQRSSSGGFGADPGLSSVRRSVSSNVFHGTSAGRPSFAPGSMSSNPA
          	    :: **.** ***:*:.    : **.**:*: .*     *:   .  : *      :

MG01027.1 	SVDNRDRRSSVYQPRAGGAAP-----SGGHQSFFQQAPQPAGVPKDPRPLKDRSYQNRIG
NCU03899.1	SQEL-DRRSSVYRPSARPSAVGGGPLAGGHMSFFQQAPQPAGVPRDPRPLKDRQFQNRIG
FG09262.1 	STDG-DRRSSVYRP--RQSTV---PAVSHHQSFFQTTPAPAGVPRDPRPLKDRSFQARIG
AN4969.1  	SQSL-QRRSSVFSRPSTGGAM-------GHQSFFTQVPSAAGVPRDPRPLRDRSFQARIA
          	* .  :*****:   :  .:         * ***  .* .****:*****:**.:* **.

MG01027.1 	QELLDYLTQHNFELDMNHNLSQNVIKSPTQKDFNYIFQWLYNRIDPSYKFMKNIDQEVPP
NCU03899.1	QELLEYLAKNNFEMEMNHKLSDNFTKSPTQKDFNYLFQWLYHRIDPSYRFQKNIDQEVPP
FG09262.1 	QEIMEYMVQHNFEMEMKHVLSQNVLKSPTQKDFNYMFQWLYHRIDPSHKFQKNIDQEVPP
AN4969.1  	QELLEYLTHNNFELEMKHSLGQNTLRSPTQKDFNYIFQWLYHRIDPGYRFQKAMDAEVPP
          	**:::*:.::***::*:* *.:*  :*********:*****:****.::* * :* ****

MG01027.1 	LLKQLRYPYEKGITKSQIAAVGGQNWSTFLGMLHWMMQLAQMIEGFALNRYADACLEAGV
NCU03899.1	LLKQLRYPYEKSITKSQIAAVGGQNWSTFLGLLHWMMQLAQMLEGYARHQYDDACMEAGV
FG09262.1 	LLKQMRYPFERSITKSQIAAVGGQNWSTFLGLLHWMMQLAQMLDGYVNCQYDEACMEAGI
AN4969.1  	ILKQLRYPYEKGITKSQIAAVGGQNWPTFLGMLHWLMELAQMMDRFAMGEYDEACAEMGV
          	:***:***:*:.**************.****:***:*:****:: :.  .* :** * *:

MG01027.1 	DTVGDHITFDFLSAAYAEWLGMDDSVSEEEADRLLKPHIEEMEAAFEQANAKHFEELRML
NCU03899.1	DIAGDQIIFDFLTSAYQDWLNMDDEAGDEDVERALQPHVQGMAEAFERSNAKYIQELEIL
FG09262.1 	DVSGDRIIFDFLSNGYRDWLAMDEDLGDEEAERVLAPHVQSMAAAFERSNSKYTSELDML
AN4969.1  	DVSGDRIIFRFLTGAYHDWLQGGEEEDDDAAAQRLIPHIELMAQEFEKGNEKYVQEMQVL
          	*  **:* * **: .* :**  .:. .:: . : * **:: *   **:.* *: .*: :*

MG01027.1 	EAENARLLKEVDDLQKSTPDPAILDQHFKIMEEDKVKFEEYNQLAIQRSEKYENRIQVLQ
NCU03899.1	EGENARLLKEIQELEKSTPDPAILDNHFKIMEEDKIKFEEYNTLAMQRTEKYENRIQVLR
FG09262.1 	EAENARLLKEIEDLEKSTPDPAVLDHHFKIMEEDKVKFEEYNALAMQRSEKYESRSQVLQ
AN4969.1  	DAENRALRDQIEELEKNAPDMAKLDKQFRILEDDKRKFEDYIQNVQGKIEKYESRIAFLE
          	:.**  * .::::*:*.:** * **::*:*:*:** ***:*   .  : ****.*  .*.

MG01027.1 	EELEKVMVELAEAEEERRSLQKAVDDQGISMQDIDRMASERERLQKTIESAGQRLEDVKR
NCU03899.1	EELGKLHVELKEAEEERRQMQKAVDDQGISMQDIDRMTSERERLQRSIESASQRLEDVKK
FG09262.1 	EELDKLLEELQEADEERRSLQKAVDAQGISMQDIDRMTAERERLQRGIESASQRLEEVKK
AN4969.1  	DEIRKTESELQAAEEERAGLQASVDQQGLTIQDIDRMNTERDRLQRSLDDAVSRLEETHA
          	:*: *   **  *:***  :* :** **:::****** :**:***: ::.* .***:.: 

MG01027.1 	KVSEKELEASQKLEELERMVDRYNTVAYQIGLIPATAVNAKGQDLELHVTVNSGPSFSGS
NCU03899.1	KVAEREMEASQRLDELERLVEKYNTVAYQIGLIPATAVNAKGKNLELQVTVNEGTPNFAS
FG09262.1 	KVSEREAEASRKLDELEQMVDRYNTMAYQIALIPSTAANAKGREFELQVTVADDSDFTTS
AN4969.1  	RVMAKESEASAKLEDLEELVKTYNTLGYQNSLIPSTAVNANGQEYELGLNVNERS-FSTS
          	:*  :* *** :*::**.:*. ***:.** .***:**.**:*:: ** :.* . .    *

MG01027.1 	SASASQLKGSYTGGSMENERLLVDPVTGYQPAHVLSLDLRGQVRSMLTSLRKEISERRGQ
NCU03899.1	SMQGVLGPNGQPLGGVENERLLADSTTGYQPAHILNLDLRGQVKNTFLALRKEVSERRKV
FG09262.1 	IMK--SGPSS-------ADRLVVDSTTGHQAGHILNLDLRGKIRNSFISLRKEISDRRAT
AN4969.1  	QIGGIPSRISP-----EADRLLAEPFTGYHPAHLLNLDLRGIVRSNLQALRKEINERRKR
          	   .     .      . :**:.:. **::..*:*.***** ::. : :****:.:**  

MG01027.1 	AMDTMMKDHDLLDSIKEAMEDKRNEVEALEHRVRAAEEEFEKTKE~VTTAQRMASDAQVE
NCU03899.1	AMDVMMEDHDLLDRAKEAIEDKRNEVEALGHRVRAAEEEYEKTKE0VTNAQKMASDAQIE
FG09262.1 	AMDEMIKDHDLLDGIKEAIEDRRGEVEALNHRVRAAEEEYEKTKE~VTTAQKLASDAHIE
AN4969.1  	GIDADLERRNLLDNIKEAMDEKRSEVEALEHKRRTAEEEFERLKE~VTTTQKLASDAQIE
          	.:*  :: ::***  ***::::*.***** *: *:****:*: ** **.:*::****::*

MG01027.1 	KMEKELAKMRASLSESVQLLEQREMTTNIE2YEQLTLKANSLREELHTEIMRMLNDVIKF
NCU03899.1	KMEKELAKMRAQLSESVQLMEQREMNTNIE2YEQLTLRANALREELHTETMRMLNDVIKF
FG09262.1 	KMEKELSRMRAGLSENVQILEQREINTTIE2YEQLVLQANALREELHTEIDRMLNDVIKF
AN4969.1  	KMEKELAKMRATMSESVQLMEQREMNTNIE~YEQLTLRANALREELHTNVESMLNDVIRF
          	******::*** :**.**::****:.*.** ****.*:**:*******:   ******:*

MG01027.1 	KMHVQKSLEEYEAFVADELEHELGSED-IMRDDDGNDVDGDS
NCU03899.1	KMHVQRSLEEYETFVADELEQELGIDDQEMEDEDTPMADV--
FG09262.1 	KIHVQKSLDDYEGFVTEELEKELGSD--EMQ-EDTQRMDM--
AN4969.1  	KVHIQKGLEDYENFVVDEVEQELGGDTQLDEDAPMSTEEL--
          	*:*:*:.*::** **.:*:*:*** : .  ..      :
```
